# *Lasiodiplodia carlesii* sp. nov. (Botryosphaeriales, Botryosphaeriaceae), a novel pathogen causing canker and dieback on *Castanopsis* (Fagaceae) plantations in China

**DOI:** 10.3897/mycokeys.137.184738

**Published:** 2026-07-21

**Authors:** Yanfeng Zhang, Jinyan Feng, Ning Jiang, Jinzhu Xu, Changsheng Qin, Fang Xu, Longyan Tian

**Affiliations:** 1 Guangdong Academy of Forestry/ Guangdong Provincial Key Laboratory of Forest Silviculture, Protection and Utilization/ Key Laboratory of National Forestry and Grassland Administration on Ecosystem Conservation and Restoration in the Guangdong-Hong Kong-Macao Greater Bay Area, Guangzhou, 510520, China Key Laboratory of Biodiversity Conservation of National Forestry and Grassland Administration, Ecology and Nature Conservation Institute, Chinese Academy of Forestry Beijing China; 2 School of Ecological Engineering, Guangdong Eco-Engineering Polytechnic, Guangzhou 510520, China Guangdong Academy of Forestry/ Guangdong Provincial Key Laboratory of Forest Silviculture, Protection and Utilization/ Key Laboratory of National Forestry and Grassland Administration on Ecosystem Conservation and Restoration in the Guangdong-Hong Kong-Macao Greater Bay Area Guangzhou China; 3 Key Laboratory of Biodiversity Conservation of National Forestry and Grassland Administration, Ecology and Nature Conservation Institute, Chinese Academy of Forestry, Beijing 100091, China School of Ecological Engineering, Guangdong Eco-Engineering Polytechnic Guangzhou China

**Keywords:** Botryosphaeriaceae, canker, forest pathology, pathogenicity, phylogenetic analysis

## Abstract

*Castanopsis*, the dominant genus in evergreen broadleaved forests, is widely distributed across South and Southeast Asia. It has significant ecological and economic value, providing resources such as timber, firewood, food, and traditional medicines. China is a center of *Castanopsis* diversity, with its plantation areas having increased steadily in recent decades. However, severe outbreaks of canker and dieback disease were observed in *Castanopsis carlesii* and *C. hystrix* plantations in 2021. In this study, we isolated fungal pathogens from diseased tissues, and identified a novel species, *Lasiodiplodia carlesii*, based on morphological characteristics and multi-locus phylogenetic analyses of ITS, *tef1-α*, *tub2*, and *rpb2*. Pathogenicity tests demonstrated that *L. carlesii* not only causes canker and dieback symptoms on multiple *Castanopsis* species (including *C. carlesii*, *C. hystrix*, and *C. faberi*), but also produces water-soaked brown lesions on the fruits of *Syzygium samarangense*. These results suggest that *L. carlesii* has a broad host range, and exhibits cross-family infection. Collectively, this study identifies *L. carlesii* as a novel pathogen posing a substantial threat to *Castanopsis* plantations and provides a critical foundation for developing targeted management strategies.

## Introduction

*Castanopsis* (D. Don) Spach (Fagaceae), a genus of evergreen trees native to South and Southeast Asia, comprises over 120 species (Huang et al. 1999; Sharma et al. 2011; Cheuk and Fischer 2021; Tang et al. 2022). These trees dominate forest canopies across diverse habitats, serving as keystone species in maintaining ecosystem function. Select species are also of significant economic value; for example, *C. piriformis* and *C. boisii* produce edible nuts, while *C. costata* is widely utilized in traditional medicine (Thang et al. 2016; Alkandahri et al. 2024). In China, approximately 60 species are distributed south of the Yangtze River. Among them, *C. carlesii* and *C. hystrix* are extensively cultivated in Guangdong and Guangxi provinces due to their rapid growth and high regenerative capacity (Lin et al. 2017; Li et al. 2023).

Despite their ecological and economic importance, *Castanopsis* plantations face increasing disease risks, a challenge often exacerbated in monoculture plantations compared to natural forests (Jactel et al. 2017). However, the pathogens affecting *C. carlesii* and *C. hystrix* remain poorly known. To date, only four foliar pathogens that typically cause limited lesions have been reported on *C. hystrix*: *Coniella granati* (red spot), *Pestalotia pauciseta* (brown spot), *Colletotrichum gloeosporioides* (anthracnose), and *Cephaleuros virescens* (algal spot) (Zhang et al. 2015). Critically, recurring stem canker and dieback symptoms have been observed in *Castanopsis* stands over the past decade, yet the etiology of these severe diseases remains uncharacterized (Sugiyama 2012; Jung et al. 2017; Jiang et al. 2025a).

Among the potential causal agents, the genus *Lasiodiplodia* Ellis & Everh. (Botryosphaeriaceae) includes prominent pathogens known to cause stem canker, dieback, and fruit rot in a wide range of subtropical and tropical woody plants (Linaldeddu et al. 2015; Huda-Shakirah et al. 2022; Hattori et al. 2023; Piattino et al. 2024; Xu et al. 2024; Zhang et al. 2024). Several species are associated with severe necrosis, such as *Lasiodiplodia rubropurpurea* on *Eucalyptus grandis* (Burgess et al. 2006), *L. citricola* on *Citrus × sinensis* (Abdollahzadeh et al. 2010), and *L. lignicola* on *Theobroma cacao* (Yang et al. 2017; Berraf-Tebbal et al. 2020). Accurate identification within this genus currently relies on a polyphasic approach combining morphology with multi-locus phylogenies of ITS, *tef1-α*, *tub2*, and *rpb2* regions (Zhang et al. 2021; Xu et al. 2024).

Given the rising incidence of dieback in Chinese *Castanopsis* plantations, there is an urgent need to clarify the causative agents. In this study, fungal isolates from symptomatic *Castanopsis* tissues were characterized through integrative taxonomy. We identified a novel species, *Lasiodiplodia carlesii*, based on multi-locus phylogeny and morphological features, and confirmed its pathogenicity, fulfilling Koch’s postulates. Furthermore, we evaluated its potential host range, demonstrating its ability to infect multiple *Castanopsis* species and cross-infect *Syzygium samarangense* (Myrtaceae). These findings significantly deepen our understanding of *Lasiodiplodia* diversity and provide a foundation for disease management in *Castanopsis* ecosystems.

## Materials and methods

### Sample collection and isolation

Symptomatic bark samples from trunks of *Castanopsis carlesii* and *C. hystrix* exhibiting canker and dieback symptoms were collected from Longan Cave Forest Farm, Guangzhou, Guangdong Province, China, on 23 April 2021. Bark fragments (approx. 10 × 10 cm) were placed in sterile paper bags, taken to the laboratory, and stored at 4 °C for fungal isolation (<48 h). Tissue segments (2 × 2 mm) were excised from the margins of lesions. After rinsing with tap water to remove residual soil particles, the segments were surface-sterilized by immersion in 75% ethanol for 5 s, followed by 3% sodium hypochlorite for 10 s, and rinsed three times in sterile distilled water. The segments were then plated onto Potato Dextrose Agar (PDA) (3 segments per plate) and incubated at 25 °C in the dark. Hyphal tips emerging from the segments were subcultured onto fresh PDA plates and incubated under identical conditions to obtain pure cultures. Fungal isolates were purified by single-spore isolation, suspended in 15% (v/v) glycerol, and cryopreserved at −70 °C. The isolates used for further study originated from the following host trees: BZ-FL-1 and BZ-FL-3 were isolated from the same *Castanopsis carlesii* tree (individual tree A), BZ-FL-11 from an independent *C. carlesii* tree (individual B), and BZ-GF-1 and BZ-GF-2 from the same *C. hystrix* (individual C). Thus, not all isolates were from independent trees. Ex-type living cultures and other obtained strains were deposited in the Guangdong Microbial Culture Collection Center (**GDMCC**), and the holotype specimens were deposited in the Herbarium of the Institute of Microbiology, Chinese Academy of Sciences (**HMAS**).

### DNA extraction, amplification, sequencing, and phylogenetic analysis

Genomic DNA was extracted from 7-day-old colonies of isolates BZ-FL-1 (GDMCC 3.1026), BZ-FL-11 (GDMCC 3.1027), BZ-GF-1 (GDMCC 3.1028), BZ-GF-2 (GDMCC 3.1029), and BZ-FL-3 grown on PDA using the CTAB (cetyltrimethylammonium bromide) method (Doyle and Doyle 1990). Partial gene sequences were generated for the internal transcribed spacer (ITS) region (including ITS1, 5.8S nrDNA, and ITS2), translation elongation factor 1-alpha (*tef1-α*), beta-tubulin (*tub2*), and the second largest subunit of RNA polymerase II (*rpb2*). Amplification was performed using the primer pairs ITS5/ITS4 (White et al. 1990), EF1-728F/EF1-986R (Carbone and Kohn 1999), Bt-2a/Bt-2b (Glass and Donaldson 1995), and RPB2-LasF/RPB2-LasR (Cruywagen et al. 2017), respectively (Table 1).

**Table 1.** Primers used in this study.

**Primer name**	**Sequences (5’-3’)**	**Reference**
ITS4	TCCTCCGCTTATTGATATGC	White et al. (1990)
ITS5	GGAAGTAAAA GTCGTAACAAGG	White et al. (1990)
EF1-728F	CATCGAGAAGTTCGAGAAGG	Carbone and Kohn (1999)
EF1-986R	TACTTGAAGGAACCCTTACC	Carbone and Kohn (1999)
Bt-2a	GGTAACCAAATCGGTGCTGCTTTC	Glass and Donaldson (1995)
Bt-2b	ACCCTCAGTGTAGTGACCCTTGGC	Glass and Donaldson (1995)
RPB2-LasF	GGTAGCGACGTCACTCCT	Cruywagen et al. (2017)
RPB2-LasR	GCGCAAATACCCAGAATCAT	Cruywagen et al. (2017)

Polymerase chain reaction (PCR) amplifications were conducted under the following thermal cycling conditions: initial denaturation at 94 °C for 5 min; followed by 32 cycles of denaturation at 94 °C for 30 s, annealing at 54 °C for 50 s, and extension at 72 °C for 1 min; and a final elongation step at 72 °C for 5 min. PCR products were visualized by electrophoresis on a 1% agarose gel (120 V, 30 min) and sequenced by Sangon Biotech (Shanghai) Co., Ltd. (Shanghai, China) using an ABI PRISM® 3730XL DNA Analyzer with the BigDye® Terminator Kit v.3.1 (Invitrogen).

ITS sequences from five representative isolates were subjected to BLASTn analysis against the NCBI GenBank nucleotide database. Validated sequences were deposited in GenBank (Accession numbers: ITS: PP152224–PP152227; *tub2*: PP188026–PP188029; *tef1-α*: PP188023–PP188025; *rpb2*: PP191127–PP191130). To determine the phylogenetic position of the newly collected isolates, a concatenated dataset of ITS, *tef1-α*, *tub2*, and *rpb2* sequences from 118 *Lasiodiplodia* strains (representing 68 species) and the outgroup *Diplodia sapinea* was compiled (Table 2). Additional reference sequences were obtained from GenBank. Alignments were performed using MEGA6, with gaps treated as missing data and ambiguous regions excluded.

**Table 2.** Strains used for phylogenetic analysis in this study.

**Species name**	**Strains**	**Host**	**Country**	**GenBank Accession No**.				**References**
				**ITS**	***tef1-α***	***tub2***	***rpb2***	
*Diplodia sapinea*	CBS 591.84	*Pinus radiata*	Chile	MT587367	MT592073	MT592530	NA	Zhang et al. (2021)
*L. acaciae*	CPC 20820*	* Acacia *	Indonesia	MT587421	MT592133	MT592613	MT592307	Zhang et al. (2021)
*L. americana*	CERC 1961*	*Pistacia vera*	United States	KP217059.1	KP217067.1	KP217075.1	NA	Chen et al. (2015)
*L. americana*	CERC 1960	*Pistacia vera*	United States	KP217058.1	KP217066.1	KP217074.1	NA	Chen et al. (2015)
*L. ananasi*	MFLUCC 23-0199*	*Ananas comosus*	Thailand	OR438376.1	NA	OR538078.1	NA	Tian et al. (2024)
*L. aquilariae*	CGMCC 3.18471*	* Aquilaria *	Laos	KY783442	KY848600	NA	KY848562	Wang et al. (2019)
*L. avicenniae*	CMW 41467*	*Avicennia marina*	South Africa	KP860835	KP860680	KP860758	KU587878	Osorio et al. (2017)
*L. avicenniae*	LAS 199	*Avicennia marina*	South Africa	KU587957	KU587947	KU587868	KU587880	Osorio et al. (2017)
*L. avicenniarum*	MFLUCC 17-2591*	*Avicennia marina*	Thailand	NR_163344.1	MK340867.1	NA	NA	Jayasiri et al. (2019)
*L. brasiliensis*	CMM 4015*	*Mangifera indica*	Brazil	JX464063	JX464049	NA	NA	Netto et al. (2014)
*L. brasiliensis*	CMW 35884	*Adansonia madagascariensis*	Madagascar	KU887094	KU886972	KU887466	KU696345	Zhang et al. (2021)
*L. bruguierae*	CBS 139638	*Bruguiera gymnorrhiza*	South Africa	KP860833	KP860678	KP860756	KU587877	Osorio et al. (2017)
*L. bruguierae*	CMW 41470*	*Bruguiera gymnorrhiza*	South Africa	KP860832	KP860677	KP860755	KU587876	Osorio et al. (2017)
*L. bruguierae*	CBS 141453	*Bruguiera gymnorrhiza*	South Africa	KP860834	KP860679	KP860757	KU587875	Osorio et al. (2017)
***L. carlesii***	**GDMCC 3.1026**	*Castanopsis* sp.	**China**	**PP152224**	**PP188023**	**PP188026**	**PP191127**	In this study
***L. carlesii***	**GDMCC 3.1027**	*Castanopsis* sp.	**China**	**PP152225**	**PP188024**	**PP188027**	**PP191128**	In this study
***L. carlesii***	**GDMCC 3.1028**	*Castanopsis* sp.	**China**	**PP152226**	**PP188025**	**PP188028**	**PP191129**	In this study
***L. carlesii***	**GDMCC 3.1029**	*Castanopsis* sp.	**China**	**PP152227**	**NA**	**PP188029**	**PP191130**	In this study
*L. chiangraiensis*	MFLUCC 21-0003*	–	Thailand	MW760853	MW815629	MW815627	NA	Wu et al. (2021)
*L. chonburiensis*	MFLUCC 16-0376*	Pandanaceae	Thailand	MH275066.1	MH412773.1	MH412742.1	NA	Tibpromma et al. (2018)
*L. chongzhouensis*	SICAUCC 23-0032*	*Juglans regia*	China	PP060648	PP061131	PP061163	NA	Wang et al. (2025)
*L. chongzhouensis*	SICAUCC 23-0033	*Juglans regia*	China	PP060649	PP061132	PP061164	NA	Wang et al. (2025)
*L. cinnamomi*	CFCC 51997*	*Cinnamomum camphora*	China	MG866028	MH236799	MH236797	MH236801	Jiang et al. (2018)
*L. cinnamomi*	CFCC 51998	*Cinnamomum camphora*	China	MG866029	MH236800	MH236798	MH236802	Jiang et al. (2018)
*L. citricola*	CBS 124706	*Citrus* sp.	Iran	GU945353	GU945339	KU887504	KU696350	Abdollahzadeh et al. (2010)
*L. citricola*	CBS 124707*	*Citrus* sp.	Iran	GU945354	GU945340	KU887505	KU696351	Abdollahzadeh et al. (2010)
*L. clavispora*	CGMCC 3.19595*	*Vaccinium uliginosum*	China	MK802165	NA	MK816338	MK809506	Wang et al. (2021)
*L. delonicis*	MFLUCC 23-0005*	*Delonix regia*	Thailand	OR052066.1	OR030466.1	OR030484.1	NA	Wu et al. (2023)
*L. endophytica*	MFLUCC 18-1121*	*Magnolia acuminata*	China	MK501838.1	MK584572.1	MK550606.1	NA	de Silva et al. (2019)
*L. euphorbiaceicola*	CMM 3609*	*Jatropha curcas*	Brazil	KF234543	KF226689	KF254926	NA	Cruywagen et al. (2017)
*L. euphorbiaceicola*	CMW 33350	*Adansonia digitata*	Botswana	KU887149	KU887026	KU887455	KU696346	Cruywagen et al. (2017)
*L. fici*	ZHKUCC 21-0125*	* Ficus *	China	ON178662.1	NA	ON599011.1	NA	Xia et al. (2022)
*L. fici*	ZHKUCC 21-0126	* Ficus *	China	ON178664.1	NA	ON599010.1	NA	Xia et al. (2022)
*L. fici*	ZHKUCC 21-0127	* Ficus *	China	ON178663.1	NA	ON599009.1	NA	Xia et al. (2022)
*L. fujianensis*	CGMCC 3.19593*	*Vaccinium uliginosum*	China	MK802164	MK887178	MK816337	MK809505	Wang et al. (2021)
*L. gilanensis*	IRAN 1523C*	*Citrus* sp.	Iran	GU945351	GU945342	KU887511	KU696357	Abdollahzadeh et al. (2010)
*L. gilanensis*	CBS 124705	*Citrus* sp.	Iran	GU945352	GU945341	KU887510	KU696356	Abdollahzadeh et al. (2010)
*L. gonubiensis*	CMW 14077*	*Syzygium cordatum*	South Africa	AY639595	DQ103566	DQ458860	KU696359	Burgess et al. (2006)
*L. gonubiensis*	CBS 116355	*Syzygium cordatum*	South Africa	AY639594	DQ103567	EU673126	KU696358	Burgess et al. (2006)
*L. gonubiensis*	CBS 138654	*Phyllanthus emblica*	Thailand	KM006443	KM006474	MT592619	MT592314	Trakunyingcharoen et al. (2015)
*L. gravistriata*	CMM 4564*	*Anacardium humile*	Brazil	KT250949	KT250950	NA	NA	Netto et al. (2017)
*L. gravistriata*	CMM 4565	*Anacardium humile*	Brazil	KT250947	KT266812	NA	NA	Netto et al. (2017)
*L. guilinensis*	CGMCC 3.20378*	*Citrus sinensis*	China	MW880672	MW884175	MW884204	MW884149	Xiao et al. (2021)
*L. henanica*	CGMCC 3.19176*	*Vaccinium uliginosum*	China	MH729351	MH729357	MH729360	MH729354	Wang et al. (2021)
*L. hormozganensis*	CBS 124708	*Mangifera indica*	Iran	GU945356	GU945344	KU887514	KU696360	Abdollahzadeh et al. (2010)
*L. hormozganensis*	IRAN 1500C*	*Olea* sp.	Iran	GU945355	GU945343	KU887515	KU696361	Abdollahzadeh et al. (2010)
*L. houttuyniae*	HGUP 24-0029*	*Houttuynia cordata* (Saururaceae)	China	PQ287134	NA	NA	NA	Sun et al. (2025)
*L. huangyanensis*	CDZM322	*Vitis vinifera*	China	OL863158	OM243809	OM228625	OM243844	Xiao et al. (2021)
*L. huangyanensis*	CGMCC 3.20380*	*Citrus reticulata*	China	MW880674	MW884177	MW884206	MW884151	Xiao et al. (2021)
*L. huangyanensis*	CGMCC 3.20381	*Citrus unshiu*	China	MW880675	MW884178	MW884207	MW884152	Xiao et al. (2021)
*L. indica*	IBP 1	angiospe	India	KM376151.1	NA	NA	NA	Prasher and Singh (2014)
*L. iraniensis*	IRAN 1520C*	*Salvadora persica*	Iran	GU945348	GU945336	KU887516	KU696363	Abdollahzadeh et al. (2010)
*L. iraniensis*	CBS 124711	*Juglans* sp.	Iran	GU945347	GU945335	KU887517	KU696362	Abdollahzadeh et al. (2010)
*L. juglandis*	SICAUCC 23-0034*	*Juglans regia*	China	PP060650	NA	PP061165	NA	Wang et al. (2025)
*L. juglandis*	SICAUCC 23-0143	*Juglans regia*	China	PP844871	NA	PP850059	NA	Wang et al. (2025)
*L. krabiensis*	MFLU 17-2617*	*Bruguiera* sp.	Thailand	MN047093.1	MN077070.1	NA	NA	Dayarathne et al. (2020)
*L. laeliocattleyae*	BOT 29	*Mangifera indica*	Egypt	JN814401	JN814428	NA	NA	Zhang et al. (2021)
*L. laeliocattleyae*	CBS 167.28*	* Laeliocattleya *	Italy	MT587425	MT592136	MT592618	MT592313	Zhang et al. (2021)
*L. lignicola*	CBS 134112*	–	Thailand	JX646797	KU887003	JX646845	KU696364	Phillips et al. (2013)
*L. liliacearum*	MFLU 24-0271*	*Dracaena* sp. (Liliaceae)	Thailand	NA	PQ683837.1	PQ683844.1	NA	Sun et al. (2025)
*L. linhaiensis*	CGMCC 3.20386*	*Citrus unshiu*	China	MW880677	MW884180	MW884209	MW884154	Xiao et al. (2021)
*L. lodoiceae*	DSM 112340*	*Lodoicea maldivica*	Mexico	MW274148	MW604230	MW604240	MW604219	Douanla-Meli (2021)
*L. macrospora*	CMM 3833*	*Jatropha curcas*	Brazil	KF234557	KF226718	KF254941	NA	Machado et al. (2014)
*L. magnoliae*	MFLUCC 18-0948*	*Magnolia candolii*	China	MK499387.1	MK568537.1	MK521587.1	NA	de Silva et al. (2019)
*L. mahajangana*	CMW 27801*	*Terminalia catappa*	Madagascar	FJ900595	FJ900641	FJ900630	KU696365	Begoude et al. (2010)
*L. mahajangana*	CBS 124926	*Terminalia catappa*	Madagascar	FJ900596	FJ900642	FJ900631	KU696366	Begoude et al. (2010)
*L. margaritacea*	CBS 122065	*Adansonia gibbosa*	Australia	EU144051	EU144066	NA	NA	Pavlic et al. (2008)
*L. margaritacea*	CBS 122519*	*Adansonia gibbosa*	Australia	EU144050	EU144065	KU887520	KU696367	Pavlic et al. (2008)
*L. marypalmiae*	CMM 2173	*Carica papaya*	Brazil	KC484839	KC481563	NA	NA	Netto et al. (2014)
*L. marypalmiae*	CMM 2275*	*Carica papaya*	Brazil	KC484843	KC481567	NA	NA	Netto et al. (2014)
*L. mediterranea*	CBS 137783*	*Quercus ilex*	Italy	KJ638312	KJ638331	KU887521	KU696368	Linaldeddu et al. (2015)
*L. mediterranea*	CBS 137784	*Vitis vinifera*	Italy	KJ638311	KJ638330	KU887522	KU696369	Linaldeddu et al. (2015)
*L. mexicanensis*	AGQMy 0014	*Chamaedorea seifrizii*	Mexico	MW274151.1	MW604234.1	MW604243.1	MW604222.1	Douanla-Meli (2021)
*L. mexicanensis*	AGQMy 0015 DSM 112342*	*Chamaedorea seifrizii*	Mexico	MW274150.1	MW604233.1	MW604242.1	MW604221.1	Douanla-Meli (2021)
*L. microconidia*	CGMCC 3.18485*	*Aquilaria crassna*	Laos	KY783441	KY848614	NA	KY848561	Wang et al. (2019)
*L. morindae*	ZHKUCC 22-0084*	*Morinda officinalis*	China	ON603984	NA	OP893932	NA	Senanayake et al. (2023)
*L. morindae*	ZHKUCC 22-0085	*Morinda officinalis*	China	ON603985	NA	OP893933	NA	Senanayake et al. (2023)
*L. nanpingensis*	CGMCC3.19596*	*Vaccinium uliginosum*	China	MK802167	NA	MK816340	MK809508	Wang et al. (2021)
*L. nanpingensis*	CGMCC3.19597	*Vaccinium uliginosum*	China	MK802168	NA	MK816341	MK809509	Wang et al. (2021)
*L. newvalleyensis*	EGY20113	*Phoenix dactylifera*	Egypt	ON392175	OP080253	OP080271	NA	El-Ganainy et al. (2022)
*L. newvalleyensis*	EGY20114 *	*Phoenix dactylifera*	Egypt	ON392180	OP080254	OP080272	NA	El-Ganainy et al. (2022)
*L. paraphysoides*	CGMCC3.19174*	*Vaccinium uliginosum*	China	MH729349	MH729355	MH729358	MH729352	Wang et al. (2021)
*L. paraphysoides*	CGMCC3.19175	*Vaccinium uliginosum*	China	MH729350	MH729356	MH729359	MH729353	Wang et al. (2021)
*L. parva*	CBS 456.78*	–	Colombia	EF622083	EF622063	KU887523	KU696372	Alves et al. (2008)
*L. parva*	CBS 494.78	–	Colombia	EF622084	EF622064	EU673114	KU696373	Alves et al. (2008)
*L. plurivora*	CBS 121103	*Vitis vinifera*	South Africa	AY343482	EF445396	KU887525	KU696375	Damm et al. (2007)
*L. plurivora*	STE-U 5803*	*Prunus salicina*	South Africa	EF445362	EF445395	KP872421	KP872479	Damm et al. (2007)
*L. ponkanicola*	CGMCC 3.20388*	*Citrus reticulata*	China	MW880685	MW884188	MW884214	MW884159	Xiao et al. (2021)
*L. pontei*	CBS 117454	*Eucalyptus urophylla*	Venezuela	MT587432	MT592144	MT592626	NA	Zhang et al. (2021)
*L. pruni*	JZB3130029*	*Prunus persica*	China	OR821993	OR831982	OR831991	NA	Zhou et al. (2024)
*L. pruni*	JZB3130030	*Prunus persica*	China	OR821994	OR831983	OR831992	NA	Zhou et al. (2024)
*L. pruni*	JZB3130031	*Prunus persica*	China	OR821995	OR831984	OR831993	NA	Zhou et al. (2024)
*L. pseudotheobromae*	CBS 116459*	*Gmelina arborea*	Costa Rica	EF622077	EF622057	EU673111	KU696376	Alves et al. (2008)
*L. pseudotheobromae*	CBS 116460	*Acacia mangium*	Costa Rica	EF622078	EF622058	KU198428	MT592322	Alves et al. (2008)
*L. regiae*	CDZM 900	*Juglans regia*	China	OK317022	OK316927	OK316947	OK316937	Wang et al. (2023b)
*L. regiae*	CDZM 911	*Juglans regia*	China	OK317023	OK316928	OK316948	OK316938	Wang et al. (2023b)
*L. regiae*	CGMCC 3.20693*	*Juglans regia*	China	OK317024	OK316929	OK316949	OK316939	Wang et al. (2023b)
*L. riauensis*	CMW54167	* Eucalyptus *	Riau	MT934415	OK172323	OK172320	OK172326	Jami et al. (2022)
*L. riauensis*	CMW54170*	* Eucalyptus *	Riau	MT934416	OK172324	OK172321	OK172327	Jami et al. (2022)
*L. riauensis*	CMW54169	* Eucalyptus *	Riau	MT934417	OK172325	OK172322	NA	Jami et al. (2022)
*L. rubropurpurea*	WAC 12535*	*Eucalyptus grandis*	Australia	DQ103553	DQ103571	EU673136	KU696380	Burgess et al. (2006)
*L. rubropurpurea*	CMW 15207	*Eucalyptus grandis*	Australia	DQ103554	DQ103572	KU887530	KU696381	Burgess et al. (2006)
*L. subglobosa*	CMM 3872*	*Jatropha curcas*	Brazil	KF234558	KF226721	KF254942	NA	Machado et al. (2014)
*L. subglobosa*	CMM 4046	*Jatropha curcas*	Brazil	KF234560	KF226723	KF254944	NA	Machado et al. (2014)
*L. syzygii*	GUCC 9719.2	*Syzygium samarangense*	Thailand	MW081991	MW087101	MW087104	NA	Meng et al. (2021)
*L. syzygii*	GUCC 9719.3	*Syzygium samarangense*	Thailand	MW081992	MW087102	MW087105	NA	Meng et al. (2021)
*L. syzygii*	GUCC 9719.1*	*Syzygium samarangense*	Thailand	MT990531	MW016943	MW014331	NA	Meng et al. (2021)
*L. thailandica*	CBS 138653	*Phyllanthus acidus*	Thailand	KM006433	KM006464	NA	NA	Trakunyingcharoen et al. (2015)
*L. thailandica*	CBS 138760*	*Mangifera indica*	Thailand	KJ193637	KJ193681	NA	NA	Trakunyingcharoen et al. (2015)
*L. theobromae*	CBS 164.96	Fruit along coral reef coast	Papua	AY640255	AY640258	KU887532	KU696383	Phillips et al. (2005)
*L. theobromae*	CBS 214.50	*Cajanus cajan*	India	MT587440	MT592152	MT592637	MT592333	Phillips et al. (2005)
*L. tropica*	CGMCC 3.18477*	*Aquilaria crassna*	Laos	KY783454	KY848616	KY848540	KY848574	Wang et al. (2019)
*L. venezuelensis*	WAC 12539*	*Acacia mangium*	Venezuela	DQ103547	DQ103568	KU887533	KU696384	Burgess et al. (2006)
*L. venezuelensis*	CMW 13512	*Acacia mangium*	Venezuela	DQ103548	DQ103569	KU887534	KP872491	Burgess et al. (2006)
*L. viticola*	UCD 2553AR*	*Vitis vinifera*	USA	HQ288227	HQ288269	HQ288306	KU696385	Úrbez-Torres et al. (2013)
*L. viticola*	CBS 128314	*Vitis vinifera*	USA	HQ288228	HQ288270	HQ288307	KU696386	Úrbez-Torres et al. (2013)
*L. vitis*	CBS 124060*	*Vitis vinifera*	Italy	KX464148	MN938928	KX464917	KX463994	Yang et al. (2017)
*L. xinyangensis*	CGMCC 3.20839*	*Vitis vinifera*	China	OL863156	OM243825	OM228638	OM243833	Wang et al. (2023a)
*L. ziziphi*	CGMCC 3.20838*	*Ziziphus jujuba*	China	OL863173	OM243826	OM228639	OM243828	Wang et al. (2023a)

Notes: The ex-type strains are indicated using “*” after strain numbers; “NA” stands for no sequence data in GenBank.

Phylogenetic analyses based on the concatenated four-locus alignment were performed utilizing both Maximum Likelihood (ML) and Bayesian Inference (BI) criteria, seamlessly implemented via the One-click Fungal Phylogenetic Tool (OFPT) v. 1.9.0 (Zeng et al. 2023). For the concatenated dataset, a gene-by-gene partitioning strategy was adopted, treating each of the four loci (ITS, TEF1-α, β-tubulin, and RPB2) as a separate partition. For the ML analyses, the best-fit nucleotide substitution models for each dataset were determined by ModelFinder, and the trees were inferred using IQ-TREE with ultrafast bootstrap approximation evaluating 1000 replicates. Bayesian Inference was executed in MrBayes v. 3.2.7 (Ronquist et al. 2012), utilizing the optimal models selected by ModelFinder under the Akaike Information Criterion (**AIC**). For each Bayesian analysis, two parallel runs, each consisting of four Markov chains (one cold, three heated), were simulated for 10 million generations, with trees sampled every 100 generations. The initial 25% of sampled trees were discarded as burn-in, and the remaining trees were utilized to calculate Bayesian posterior probabilities (BPP). Convergence was confirmed when the average standard deviation of split frequencies dropped below 0.01. The resulting phylogenetic trees were visualized and graphically edited in FigTree v. 1.4.2.

### Morphological studies

Colony morphology of isolate BZ-FL-1 was characterized on Potato Dextrose Agar (**PDA**), 2% Malt Extract Agar (**MEA**), Synthetic Nutrient-Poor Agar (**SNA**), and Czapek Dox Agar (**CDA**) after incubation at 25 °C in the dark for one week. To induce sporulation, isolates were cultured on PDA, MEA, and Pine Needle Agar (**PNA**) supplemented with sterile 5-cm-long twigs of *Castanopsis carlesii*, and maintained at 25 °C under continuous near-UV light for 2–4 weeks (Zhang et al. 2021; Xu et al. 2024). Morphological characteristics of conidiogenous cells, conidiomata, conidiophores, and paraphyses were examined using a Zeiss SteREO Discovery.V20 dissecting microscope and a Nikon ECLIPSE Ni-U compound microscope equipped with differential interference contrast (**DIC**) optics. Both microscopes were coupled with a Nikon DS-Fi3 digital camera. Conidial dimensions were determined by measuring 200 mature conidia grown on PDA (with or without *C. carlesii* twigs). The average length and width, standard deviation (**SD**), and minimum/maximum dimensions were calculated. Data are presented as: length × width = (min–) mean ± SD (–max) × (min–) mean ± SD (–max). The length/width ratio (L/W) was also calculated.

### Pathogenicity tests

Pathogenicity tests were conducted on healthy two-year-old seedlings of *C. carlesii*, *C. faberi* and *C. hystrix*. Shoots (approx. 1.0 cm diameter) were surface-sterilized with 75% ethanol and rinsed with sterile water. Three wounds were created on each seedling using a sterile 5-mm cork borer. Wounds were inoculated with 5-mm mycelial plugs excised from the margins of actively growing 3-day-old PDA cultures of isolates BZ-FL-1 or BZ-FL-3. The control groups (CK) were designated as CK-C, CK-F, CK-H, and the treatment were designated as 1C, 1F, 1H, 3C, 3F, 3H. Inoculation sites were wrapped with wet sterile gauze for 24 h to maintain humidity (Chen et al. 2011; Wang et al. 2023b). Wounds inoculated with sterile PDA plugs served as negative controls. All seedlings were maintained in a greenhouse at 25 ± 2 °C, 75% ± 5% relative humidity, with a photoperiod of 12 h light / 12 h dark. Lesion development was monitored, and lesion length (longitudinal) was measured using digital calipers at 3, 7, and 10 days post-inoculation (dpi), respectively. To fulfill Koch’s postulates, fungi were re-isolated from the lesions and identified via morphological characteristics and sequencing of the ITS, *tef1-α*, and *tub2* regions as described above. Each treatment included ten replicates, and the entire experiment was repeated twice.

To further assess virulence and host range, healthy fruits of *S. samarangense* were inoculated with mycelial plugs from four strains (BZ-FL-1, BZ-FL-11, BZ-GF-1, and BZ-GF-2), following the method of Meng et al. (2021) with minor modifications. Each treatment included six replicates, and the experiment was repeated twice. Strains re-isolated from lesion margins of *S. samarangense* were confirmed by the same morphological and molecular methods used previously.

### Statistical analysis

The lesion length data from the nine groups (CK-C, CK-H, CK-F, 1C, 1F, 1H, 3C, 3F, 3H) were first assessed for normality using the Shapiro–Wilk test. Given the violation of normality, a Kruskal-Wallis H test followed by Dunn’s post-hoc test with Bonferroni correction was performed. Exact adjusted p-values are reported.

## Results

### Fungal isolates

**Figure 1.**
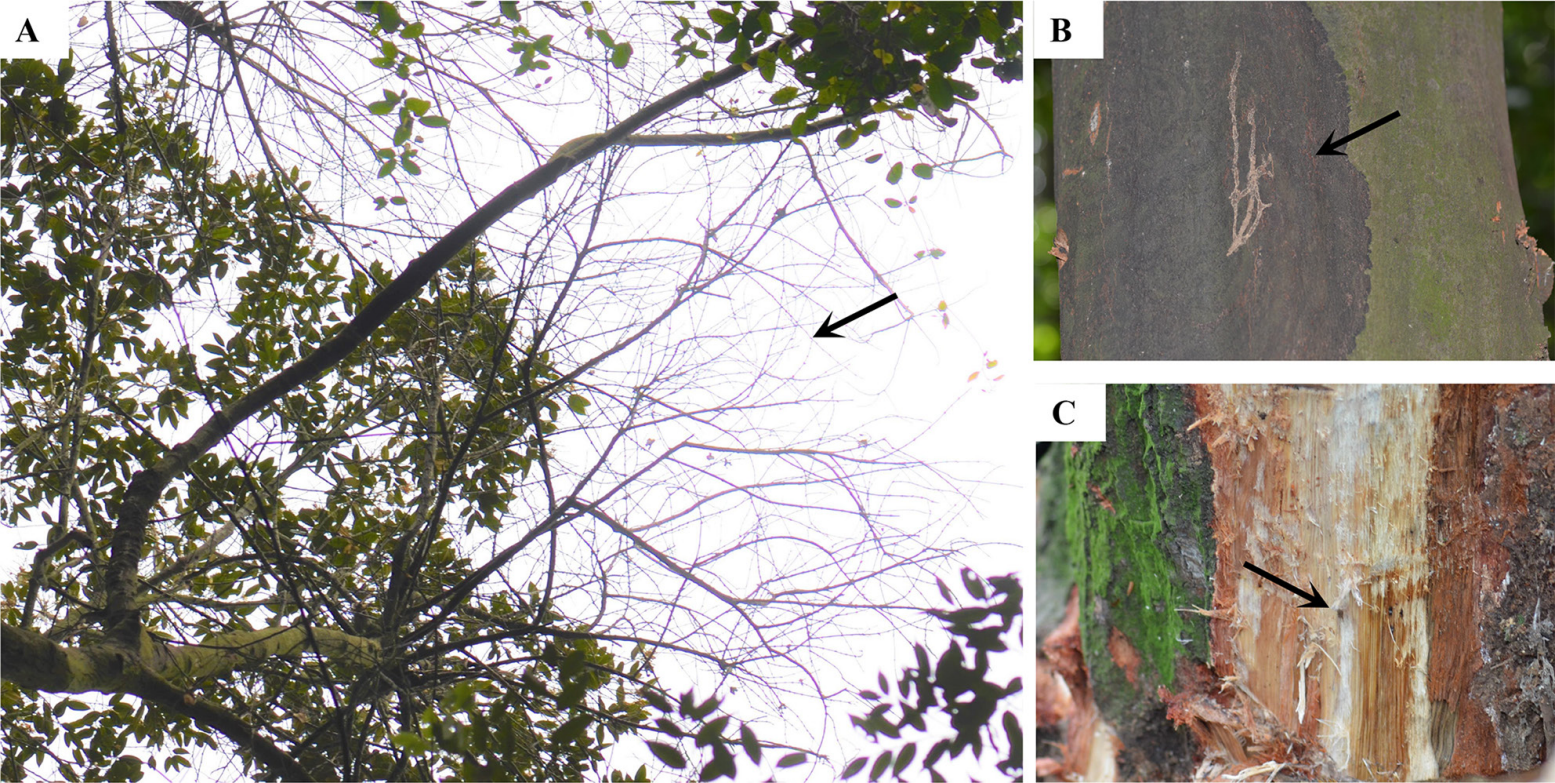
Symptoms (indicated by the arrows) on *Castanopsis carlesii*. **A**. Canopy dieback; **B**. Outer bark necrosis; **C**. Necrosis on the trunk base.

A total of 20 fungal strains were isolated from canker and dieback lesions on *Castanopsis carlesii* and *C. hystrix* (Fig. 1). Preliminary BLASTn analysis of the ITS region revealed that 15 strains (represented by BZ-FL-1, BZ-GF-1, and BZ-GF-2) shared 98% identity (98% query coverage) with the ex-type strain of *Lasiodiplodia theobromae* (CBS 164.96). One strain (BZ-FL-11) showed 99% identity (89% query coverage; E value < 1e-10) to the ex-type strain of *Lasiodiplodia rubropurpurea* (WAC 12535). These 16 *Lasiodiplodia*-like strains produced conidia that were initially hyaline and aseptate, becoming dark brown, 1-septate, and longitudinally striated upon maturity, consistent with the typical morphology of *Lasiodiplodia* species. The remaining four strains, including BZ-FL-3, shared 99% identity (97% query coverage) with the ex-type strain of *Pseudofusicoccum calophylli* (MFLUCC 17-2533).

### Pathogenicity on *Castanopsis* species

**Figure 2.**
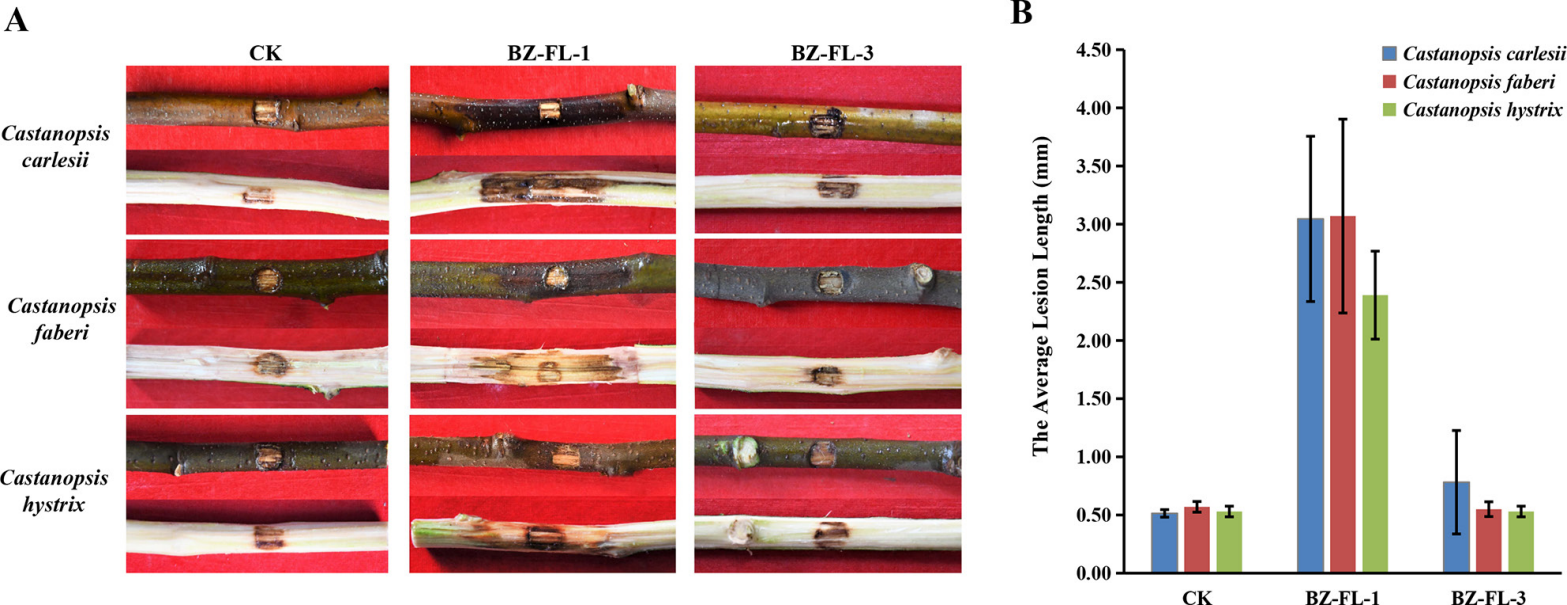
Pathogenicity test on *Castanopsis carlesii*, *C. faberi*, and *C. hystrix* branches inoculated with *Lasiodiplodia* sp. strain BZ-FL-1 or sterile PDA plugs (control, CK) at 7 dpi. **A**. Disease symptoms; **B**. Lesion size (mean ± SD). n = 10 inoculation points per treatment.

To identify the causal agent of canker and dieback on *Castanopsis* species, representative isolates BZ-FL-1 (*Lasiodiplodia* sp.) and BZ-FL-3 (*Pseudofusicoccum* sp.) were selected randomly for pathogenicity assays. In the *in vivo* inoculation tests, only isolate BZ-FL-1 induced dark brown bark rot and necrosis around the inoculation sites on *C. carlesii*, *C. faberi*, and *C. hystrix* within 3 dpi, with lesion expansion becoming evident by 7 dpi (Fig. 2A). Dieback symptoms were observed in three out of twenty *C. carlesii* seedlings inoculated with BZ-FL-1; notably, these symptoms developed only after the necrotic lesions had completely girdled the stems (approx. 30 dpi). In contrast, shoots inoculated with *Pseudofusicoccum* sp. BZ-FL-3 remained asymptomatic, similar to the negative controls (Fig. 2A). The pathogen was successfully re-isolated from the necrotic tissues and confirmed as *Lasiodiplodia* sp. BZ-FL-1 based on morphological characteristics and ITS sequence analysis (98% identity to *L. theobromae* CBS 164.96), thereby fulfilling Koch’s postulates.

On all three tested *Castanopsis* species, Kruskal-Wallis tests revealed highly significant differences in lesion length among the three groups (CK, BZ-FL-1, BZ-FL-3) (*C. carlesii*: χ^2^(2) = 22.388, *p* < 0.001; *C. faberi*: χ^2^(2) = 19.631, *p* < 0.001; *C. hystrix*: χ^2^(2) = 21.805, *p* < 0.001). Dunn’s post-hoc tests with Bonferroni correction showed that, on all three species, BZ-FL-1 produced significantly larger lesions than both CK and BZ-FL-3 (adjusted *p* < 0.01), whereas no significant differences were detected between BZ-FL-3 and CK (adjusted *p* > 0.05). Thus, BZ-FL-1 is highly pathogenic to all three *Castanopsis* species tested, while BZ-FL-3 exhibits no pathogenic effect. The Kruskal-Wallis test showed no statistically significant difference in lesion length among the three tree species induced by *Lasiodiplodia* sp. BZ-FL-1 (χ^2^(2) = 4.446, *p* = 0.108). Although the mean rank is highest for *C. carlesii* (19.65), followed by *C. faberi* (16.91) and *C. hystrix* (11.35), the differences do not reach significance at α = 0.05 (Suppl. material 1: tables S1, S2).

### Pathogenicity on *Syzygium samarangense*

**Figure 3.**
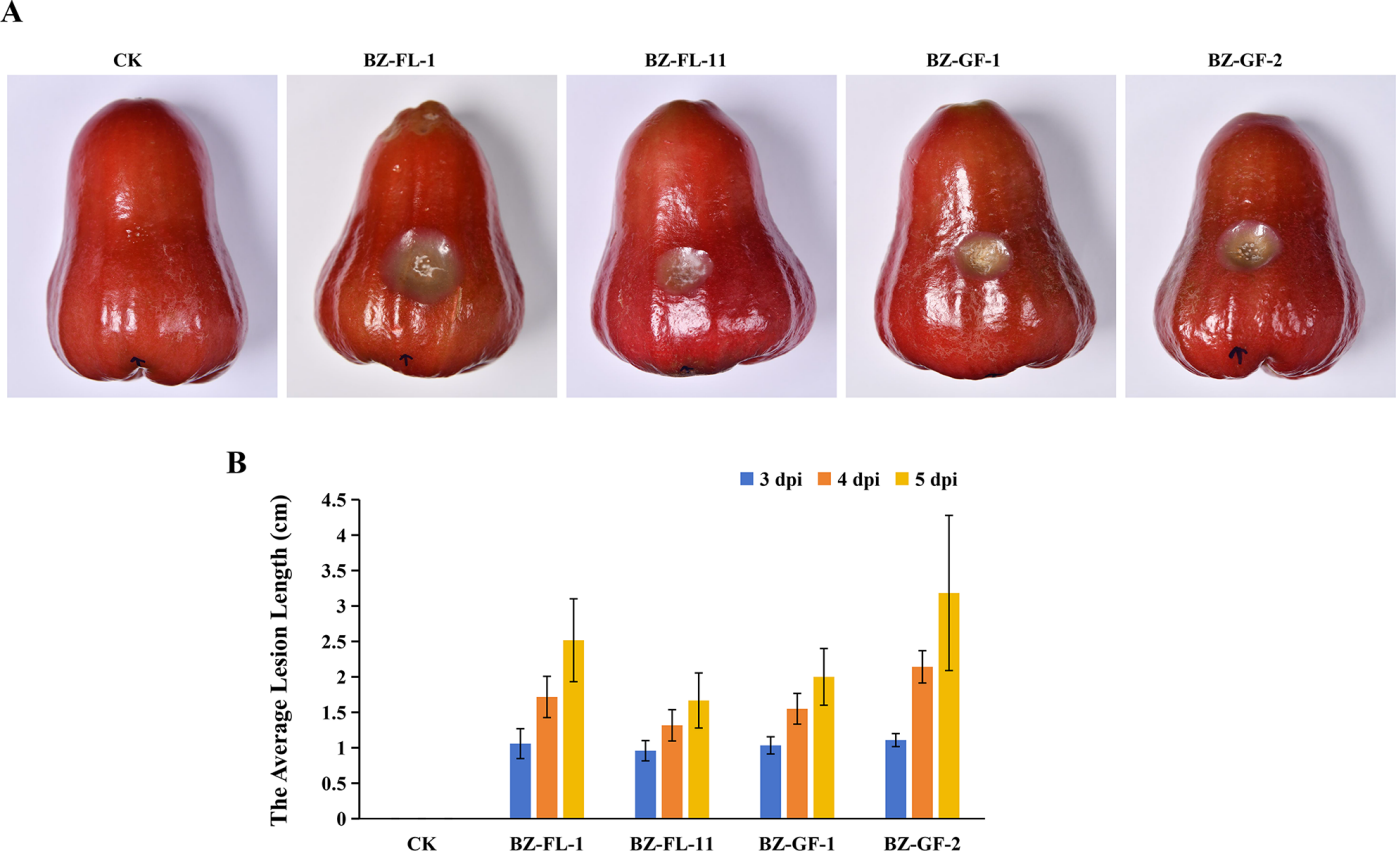
Pathogenicity test on *Syzygium samarangense* berries inoculated with *L. carlesii* strains BZ-FL-1, BZ-FL-11, BZ-GF-1, BZ-GF-2 or sterile PDA plugs (CK) at 3 dpi. **A**. Disease symptoms; **B**. Lesion size. n = 6 inoculation points per treatment.

Inoculation assays on *S. samarangense* revealed that isolates BZ-FL-1, BZ-FL-11, BZ-GF-1, and BZ-GF-2 all induced water-soaked brown lesions on the fruit tissues (Fig. 3). Lesion diameters were measured at 3, 4, and 5 dpi. Kruskal-Wallis tests revealed significant differences among treatments at all time points (3 dpi: χ^2^(4) = 16.545, *p* = 0.002; 4 dpi: χ^2^(4) = 22.634, *p* < 0.001; 5 dpi: χ^2^(4) = 21.595, *p* < 0.001). Dunn’s post-hoc test (Bonferroni correction) showed that BZ-FL-1 and BZ-GF-2 produced significantly larger lesions than CK at 3, 4, and 5 dpi (adjusted *p* ≤ 0.013 and *p* ≤ 0.003, respectively); BZ-GF-1 was significantly larger than CK only at 3 dpi (*p* = 0.046); BZ-FL-11 was never significantly different from CK (*p* > 0.05) (Suppl. material 1: tables S3, S4). Among strains, only BZ-FL-11 vs BZ-GF-2 at 4 dpi was significant (*p* = 0.046); no other pairwise comparisons were significant. Consequently, BZ-FL-1 and BZ-GF-2 displayed strong and persistent pathogenicity, significantly enlarging lesions at all three time points. Given the small sample size (n = 6) and the conservative nature of Bonferroni adjustment, BZ-GF-1 showed a transient effect only at 3 dpi, while BZ-FL-11 exhibited no significant pathogenic effect at any time point. These two strains possess weak but detectable pathogenicity.

### Phylogenetic analyses

**Figure 4.**
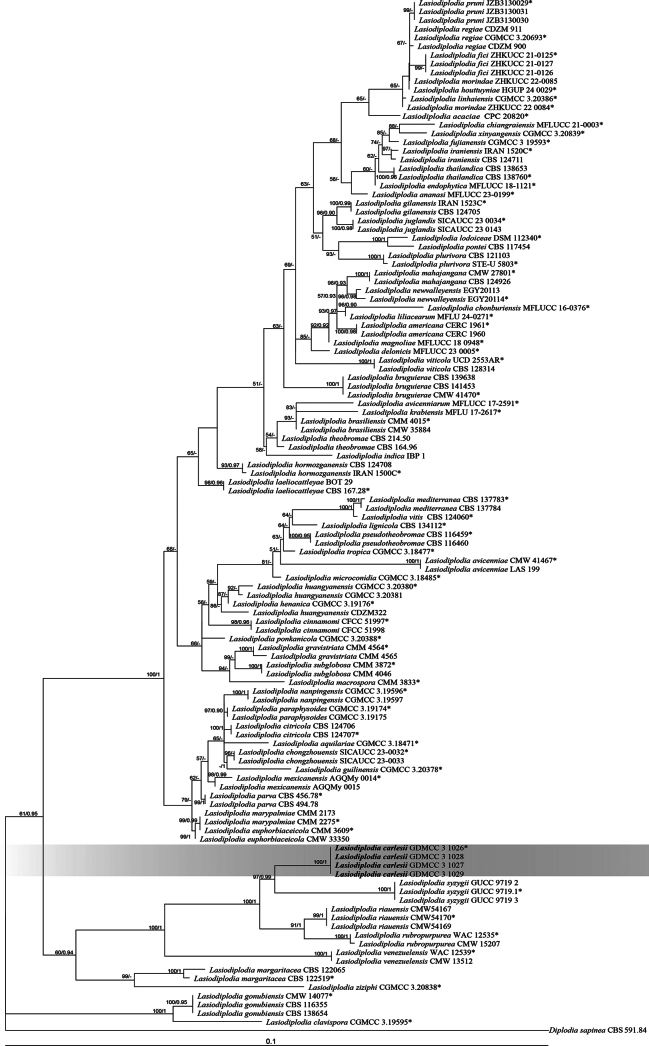
Phylogenetic analysis of *Lasiodiplodia* based on the combined ITS, *tef1-α*, *tub2* and *rpb2* sequences data. Numbers above the branches indicate ML bootstrap values (ML-BS ≥ 50%) and Bayesian Posterior Probabilities (BPP ≥ 0.9). The tree is rooted with *Diplodia sapinea* (CBS 591.84). The newly identified species, *Lasiodiplodia carlesii*, is denoted in gray, and ex-type strains are marked with “*”.

The concatenated dataset of ITS, *tef1-α*, *tub2*, and *rpb2* sequences for the *Lasiodiplodia* analysis comprised 1,782 aligned characters (including gaps), with partition lengths of 436 bp (ITS), 446 bp (*tef1-α*), 375 bp (*tub2*), and 522 bp (*rpb2*). The optimal nucleotide substitution models selected for the respective loci were K2P+G4 for ITS, TNe+G4 for *rpb2*, TNe+R3 for *tef1-α*, and TN+F+G4 for *tub2*. The Maximum Likelihood (ML) analysis generated a best-scoring tree with an optimization likelihood value of –8006.63. The alignment matrix contained 502 distinct site patterns, with 22.84% consisting of undetermined characters or gaps. The estimated empirical base frequencies were A = 0.218444, C = 0.294418, G = 0.264566, and T = 0.222572. The substitution rates were calculated as AC = 1.123564, AG = 4.378672, AT = 1.499785, CG = 1.038398, CT = 7.051007, and GT = 1.0000. The gamma distribution shape parameter (α) was estimated at 0.184441. The overall tree topology generated from the ML analysis was highly congruent with that of the Bayesian Inference (BI) consensus tree (Fig. 4).

In the phylogenetic tree, the four isolates (BZ-FL-1, BZ-FL-11, BZ-GF-1, and BZ-GF-2) formed a distinct, strongly supported monophyletic clade (ML/BI = 100/1; Fig. 4). This clade is phylogenetically sister to *Lasiodiplodia syzygii*. However, the new species differs from *L. syzygii* by specific nucleotide substitutions: 6 bp in ITS, 5 bp in *tef1-α*, and 3 bp in *tub2* (Table 3).

**Table 3.** Changes in pair base of *L. carlesii* and *L. syzygii* in the ITS, *tef1-α* and *rpb*2 genes.

**Genes**	**Alignment position**	**Base pair difference**		**Location**
		***L. syzygii* (5 isolates)**	***L. carlesii* (4 isolates)**	
ITS	468	T	C	48
		T	C	50
		C	T	98
		T	C	130
		C	T	173
		C	T	362
*tef1*	223	A	G	2
		G	T	7
		T	C	175
		C	T	182
		A	G	187
*tub2*	395	C	T	209
		C	A	218
		T	C	351

### Taxonomy

#### Lasiodiplodia carlesii

Taxon classificationFungiBotryosphaerialesBotryosphaeriaceae

L.Y. Tian, Y.F. Zhang & Ning Jiang sp. nov.

537E4888-EA80-5045-AE4C-FB5CA39FBF4C

861827

Fig. 5.

##### Etymology.

In reference to the host species *Castanopsis carlesii*.

**Figure 5.**
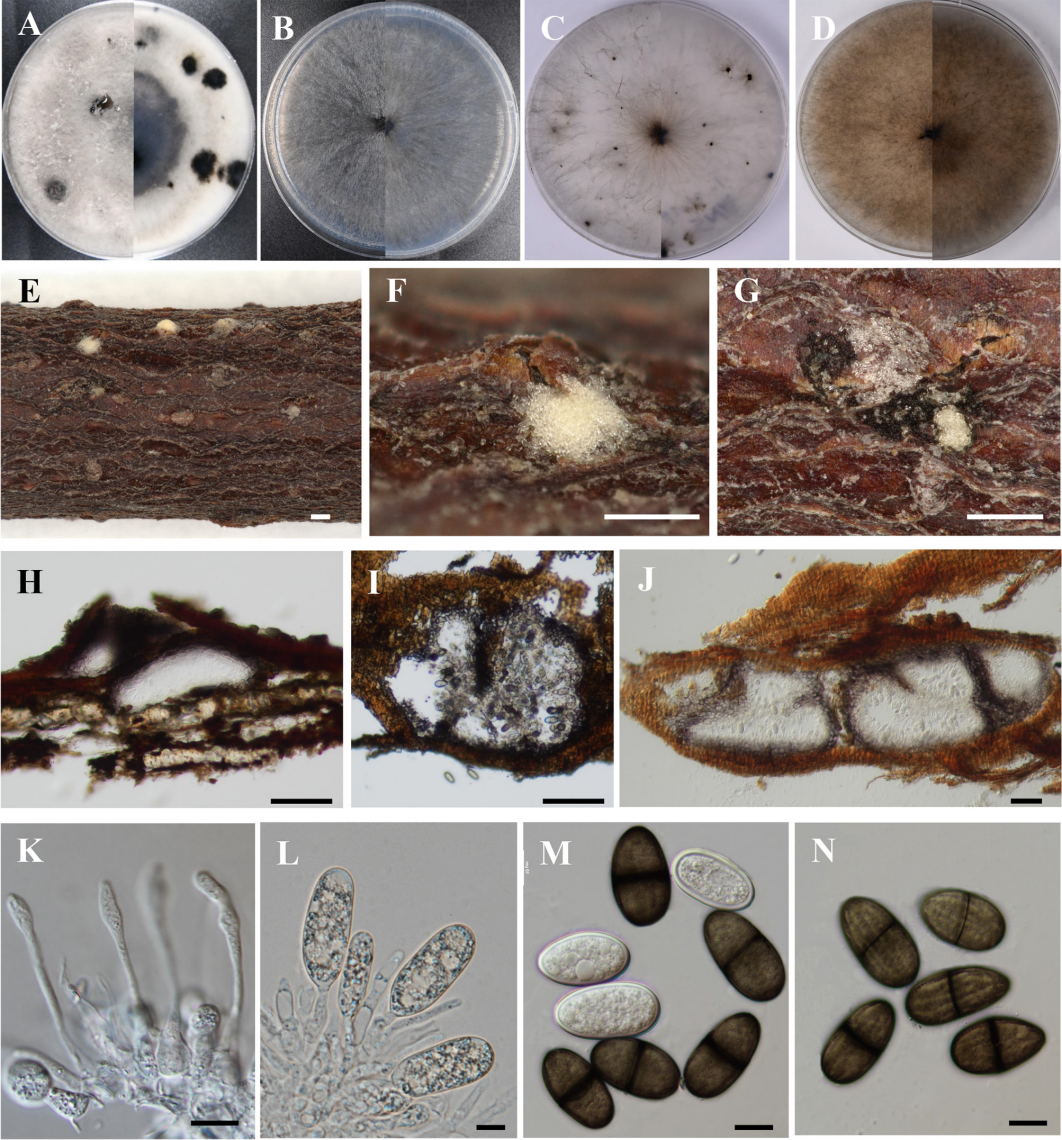
Morphological characteristics of *Lasiodiplodia carlesii* (HMAS 353197). **A–D**. Colonies on PDA, MEA, SNA, and Czapek after 15 days at 25 °C in the dark (the left and right halves of each picture showed surface and reverse sides, respectively); **E–G**. Conidiomata formed on *C. carlesii* shoots; **H**. Transverse section; **I, J**. Cross sections of conidioma; **K**. Paraphyses; **L**. Conidiophores and conidiogenous cells; **M, N**. Hyaline immature and brown mature conidia. Scale bars: 250 μm **(E**–**G)**; 100 μm **(H**–**J)**; 10 μm **(K**–**N**).

##### Typification.

China • Guangdong Province: Guangzhou City, Tianhe District, Longan Cave Forest Farm (23°14.80'N, 113°23.40'E, alt. 176 m), from trunk of *Castanopsis carlesii* (Hemsl.), on 23 April 2021, L.Y. Tian (Holotype HMAS 353197, ex-type living culture GDMCC 3.1026); • Guangdong, Guangzhou City, Longan Cave Forest Farm (23°13.80'N, 113°23.40'E, alt. 176 m), on necrotic trunk of *C. carlesii*, on 23 April 2021, L.Y. Tian (Paratype HMAS 353198, ex-paratype living culture GDMCC 3.1027).

##### Other specimens examined.

China • Guangdong, Guangzhou City, Longan Cave Forest Farm (23°13.80'N, 113°23.40'E, alt. 247 m), on necrotic trunk base of *Castanopsis hystrix*, on 23 April 2021, L.Y. Tian (HMAS 353199, living culture GDMCC 3.1028; HMAS 353200, living culture GDMCC 3.1029).

##### Description.

Asexual morph on PDA and *Castanopsis carlesii* twigs: ***Conidiomata*** stromatic, dark brown to black, solitary, semi-immersed to superficial, unilocular, papillate or apapillate, covered by dark brown mycelium; 530.1 ± 279.5 × 395.8 ± 145.2 μm, 292.2 ± 213.9 μm high (n = 20). ***Paraphyses*** interspersed with conidiophores, filiform, hyaline, cylindrical with swollen rounded apices, initially aseptate, becoming 1–7-septate at maturity, unbranched, extending beyond the conidial layer, up to 59.8 ± 12.0 μm long and 3.2 ± 1.1 μm wide. ***Conidiophores*** absent or reduced to conidiogenous cells. ***Conidiogenous cells*** holoblastic, hyaline, discrete, smooth, thin-walled, cylindrical to ampulliform, proliferating percurrently, (4.0–) 8.3 ± 2.2 (–12.5) × (1.7–) 3.9 ± 1.1 (–6.9) μm (n = 20). ***Conidia*** initially hyaline, aseptate, thick-walled (wall 1.5–3.2 μm thick), ellipsoid to ovoid with rounded or slightly tapered apices, becoming dark brown, 1-septate with longitudinal striations at maturity; (20.1–) 28.1 ± 2.9 (–33.5) × (9.9–) 14.5 ± 1.7 (–16.9) μm (mean ± SD = 28.1 ± 2.9 × 14.5 ± 1.7 μm, n = 200, L/W = 1.9). Conidial dimensions vary on different substrates: on PDA: 25.9 ± 3.6 × 12.5 ± 1.2 μm (n = 50, L/W = 2.1); on inoculated host branches: 28.9 ± 2.1 × 15.4 ± 0.9 μm (n = 50, L/W = 1.9).

##### Cultural characteristics.

Colonies on PDA initially white, turning gray with age, floccose with entire margins, covering the 90 mm Petri dish after 48 h at 25 °C in the dark; reverse dark gray with black patches; sporulation sparse. On MEA, colonies initially white, becoming fluffy with entire margins, covering the dish after 4 days at 25 °C; reverse dark gray to yellowish with a rust tint upon aging. On SNA, aerial mycelium scant and fluffy, initially white, becoming gray with age, with entire margins, reaching the edge of the 90 mm dish after 4 days at 25 °C; reverse gray with black patches; sporulation not observed. On Czapek Dox Agar, colonies initially white, becoming gray with gray-brown tones, fluffy with entire margins, covering the dish after 4 days at 25 °C.

##### Notes.

Phylogenetically, *Lasiodiplodia carlesii* forms a distinct and highly supported clade based on the concatenated gene alignment. It is most closely related to *L. syzygii* but differs by distinct nucleotide substitutions: 6 bp in ITS, 5 bp in *tef1-α*, and 3 bp in *tub2* (Table 3). Morphologically, *L. carlesii* can be distinguished from *L. syzygii* by its smaller conidia [(20.1–) 25.2–31.0 (–33.5) × (9.9–) 12.8–16.2 (–16.9) μm (av. = 28.1 × 14.5 μm, L/W = 1.9)] compared to those of *L. syzygii* [(24.5–) 28.0–32.5 (–39.5) × (13–) 15–17 (–21) μm (av. = 30.2 × 16.1 μm, L/W = 1.9)] (Zhang et al. 2021) (Table 4). Both species also differ in cultural characteristics: *L. carlesii* colonies cover 90 mm PDA plates with regular margins within 3 days at 25 °C, whereas *L. syzygii* requires 4 days for full coverage and exhibits irregular margins (Zhang et al. 2021). These combined phylogenetic, morphological, and biological differences support the establishment of *L. carlesii* as a new species.

**Table 4.** A comparative analysis of conidial dimensions among *Lasiodiplodia carlesii* and its phylogenetically closest relatives.

**Identity**	**Hosts**	**Conidial size (μm)**	**L/W**	**Paraphyses**	**Reference**
*L. carlesii*	*Castanopsis* spp.	(20.1–)25.2–31.0(–33.5) × (9.9–)12.8–16.2(–16.9) [Av. 28.1 × 14.5]	1.9	Septatae	This study
*L. syzygii*	*Syzygium samarangense*	(27–)30–32(–36) × (13–)15–17(–20) [Av. 31.3 × 16.4]	1.9	Aseptatae	Meng et al. (2021)
*L. rubropurpurea*	*Eucalyptus grandis*	24–33 × 13–17 [Av. 28.2 × 14.6]	1.9	Aseptatae	Burgess et al. (2006)
*L. venezuelensis*	*Acacia mangium*	26–33 × 12–15 [Av. 28.4 × 13.5]	2.1	Septatae	Burgess et al. (2006)

## Discussion

In this study, we identified and described *Lasiodiplodia carlesii* as a novel pathogen causing canker and dieback in *Castanopsis* species. Following recent global taxonomic revisions, the genus *Lasiodiplodia* currently comprises 34 recognized species associated with plants spanning 54 genera and 39 families in subtropical and tropical regions (Zhang et al. 2021). Notably, despite this broad host spectrum, records within the Fagaceae remain rare; only two species, *L. mahajangana* (syn. *L. exigua*) and *L. mediterranea*, have been documented on *Quercus ilex* (Linaldeddu et al. 2015; Jiang et al. 2025b). To our knowledge, this is the first report of *Lasiodiplodia* species infecting *Castanopsis*, thereby filling a gap in the known host range of this genus.

The plurivorous nature of *Lasiodiplodia* is well documented (Batista et al. 2021; Gómez et al. 2021). For example, the cosmopolitan species *L. theobromae* causes Botryosphaeria canker on grapevine (*Vitis vinifera*) (Úrbez-Torres 2011), stem-end rot on papaya (*Carica papaya*) (de Silva et al. 2019), and vascular streak dieback on cacao (*Theobroma cacao*) (Ali et al. 2020). Similarly, *L. regiae* and *L. pseudotheobromae* have been reported to cause canker and dieback on a wide array of distantly related hosts, including *Juglans*, *Actinidia*, *Ziziphus*, and *Celtis* (Wang et al. 2021; Zhang et al. 2022; Guo et al. 2023; Wang et al. 2023b; Jiang et al. 2025c). Consistent with this genus-wide trait, *L. carlesii* induced symptoms not only on the three tested *Castanopsis* species (Fagaceae), but also on *S. samarangense*, the host of its phylogenetic sister species, *L. syzygii*.

However, the pathogenicity tests on *S. samarangense* were conducted using detached fruits under controlled laboratory conditions. This approach does not account for factors that influence disease development in nature, including host physiological defense responses, wound healing dynamics, microclimate variation, or interactions with epiphytic or endophytic microorganisms. Therefore, the results should be interpreted as indicating pathogenic potential rather than definitive field pathogenicity. Further studies employing whole-plant inoculations under field or semi-field conditions are needed to confirm cross-family infectivity.

The historically ambiguous taxonomy of *Lasiodiplodia* has been largely resolved through comprehensive multi-locus phylogeny (Dissanayake et al. 2016; Crous et al. 2019; Hattori et al. 2023; Wang et al. 2023a). While phylogenies based on ITS, *tef1-α*, *tub2*, and *rpb2* loci provide the primary framework for species delineation, morphological traits (particularly conidial dimensions and paraphyses) remain valuable diagnostic characters (Zhang et al. 2021). However, our study revealed significant phenotypic plasticity in *L. carlesii* conidia across different environments (PDA: 25.9 × 12.5 μm, L/W = 2.1 vs. host tissue: 28.9 × 15.4 μm, L/W = 1.9). This observation aligns with the findings of Xu et al. (2024), who reported substantial intraspecific variation in conidial dimensions among isolates of *L. euphorbiaceicola*, *L. mahajangana*, and *L. pseudotheobromae*. These findings highlight the sensitivity of conidial morphology to growth conditions and underscore the necessity of integrating stable molecular markers with standardized morphological protocols (e.g., specific media, culture age, and temperature) to minimize misclassification.

This study identifies *L. carlesii* as the causal agent of severe canker and dieback on *C. carlesii*, *C. faberi*, and *C. hystrix*, thereby expanding the known host range of *Lasiodiplodia* species. The pathogen also infects at least one host outside the Fagaceae, indicating a risk of spillover in mixed plantations. Moreover, abundant fruiting bodies develop at infection sites on *Castanopsis* during the late inoculation stage, suggesting a potential source of secondary inoculum. Accordingly, regular monitoring for canker and dieback symptoms should be reinforced, particularly on *Castanopsis* and adjacent non-Fagaceae trees. Infected plant material should be promptly removed, and cross-stand movement of seedlings, cuttings, or pruning tools must be avoided. An integrated disease management strategy is recommended, including the use of pathogen-free planting stock, silvicultural practices that minimize mechanical wounds, and further research on fungicide sensitivity and biological control agents.

In conclusion, this study identifies *L. carlesii* as the etiological agent of severe canker and dieback in *Castanopsis carlesii*, *C. faberi*, and *C. hystrix*, thereby expanding the host range of *Lasiodiplodia* species and providing a foundational basis for the development of disease management strategies in *Castanopsis* plantations.

## Supplementary Material

XML Treatment for Lasiodiplodia carlesii

## Additional information

### Artificial Intelligence (AI) use

The authors accept full responsibility for the content of the manuscript, including the disclosure of any use of AI.

No AI tools were used in the preparation of this manuscript.

### Author ORCIDs

Y. Zhang https://orcid.org/0000-0003-1698-2339

N. Jiang https://orcid.org/0000-0002-9656-8500

J. Xu https://orcid.org/0000-0002-9720-3861

C. Qin https://orcid.org/0000-0002-1521-6627

F. Xu https://orcid.org/0000-0002-6150-729X

L. Tian https://orcid.org/0000-0002-7213-7204

### Data availability

The datasets generated and analyzed during the current study are available in the NCBI repository with the accession numbers (PP191127; PP188027; PP188024; PP191128; PP191128; PP188026; PP188023; PP191127; PP191129; PP188028; PP188025; PP191129; PP191130; PP188029; PP191130).

## Supplementary materials

Supplementary material 1 Pathogenecity test data Data type docx https://binary.pensoft.net/file/1721059This dataset is made available under the Open Database License (http://opendatacommons.org/licenses/odbl/1.0/). The Open Database License (ODbL) is a license agreement intended to allow users to freely share, modify, and use this Dataset while maintaining this same freedom for others, provided that the original source and author(s) are credited. Yanfeng Zhang, Jinyan Feng, Ning Jiang, Jinzhu Xu, Changsheng Qin, Fang Xu, Longyan Tian
