# Supplementary material for: Lasiodiplodia carlesii sp. nov. (Botryosphaeriales, Botryosphaeriaceae), a novel pathogen causing canker and dieback on Castanopsis (Fagaceae) plantations in China
Source: MycoKeys. 2026 Jul 21;137:81–102. doi: 10.3897/mycokeys.137.184738 (PMC13416940; doi:10.3897/mycokeys.137.184738)
Supplement: Supplementary material 1 — Pathogenecity test data [file mycokeys-137-081-s001.docx]

Supplementary Table 1. Median (interquartile range) lesion lengths (cm) on three *Castanopsis* species inoculated with CK, BZ‑FL‑1, and BZ‑FL‑3.

| Host species | CK | BZ‑FL‑1 | BZ‑FL‑3 |
| --- | --- | --- | --- |
| *C. carlesii* | 0.50 (0.00) | 3.20 (1.50) | 0.60 (0.70) |
| *C. faberi* | 0.60 (0.10) | 3.25 (2.00) | 0.53 (0.10) |
| *C. hystrix* | 0.50 (0.00) | 2.15 (0.40) | 0.50 (0.00) |

Note: n=10 per treatment per host. Data were recorded at 7 days post‑inoculation. IQR = interquartile range (Q3–Q1). CK and BZ‑FL‑3 produced very small or zero lesions, resulting in IQR = 0 or near 0 for most of those groups.

Supplementary Table 2. Statistical comparison of lesion lengths among 2 strains on three *Castanopsis* species. Data are from Kruskal‑Wallis tests followed by Dunn’s post‑hoc tests (Bonferroni correction). Adjusted *p*‑values are shown for pairwise comparisons.

| Host species | Overall χ² (df=2) | *p*‑value | Pairwise comparisons (adjusted *p*) |
| --- | --- | --- | --- |
| *C. carlesii* | 22.388 | < 0.001 | BZ‑FL‑1 > CK (< 0.001); BZ‑FL‑1 > BZ‑FL‑3 (0.003); CK vs BZ‑FL‑3 (0.593) |
| *C. faberi* | 19.631 | < 0.001 | BZ‑FL‑1 > CK (0.002); BZ‑FL‑1 > BZ‑FL‑3 (< 0.001); CK vs BZ‑FL‑3 (1.000) |
| *C. hystrix* | 21.805 | < 0.001 | BZ‑FL‑1 > CK (< 0.001); BZ‑FL‑1 > BZ‑FL‑3 (< 0.001); CK vs BZ‑FL‑3 (1.000) |

Note: “>” indicates significantly larger lesion length. Bonferroni‑adjusted *p* < 0.05 considered significant.

Supplementary Table 3. Descriptive statistics of lesion diameters (cm) inoculated with BZ-FL‑1, BZ-FL‑11, BZ-GF‑1, and BZ-GF‑2 on *Syzygium* samarangense at 3, 4, and 5 days post‑inoculation (dpi).

| Treatment | 3 dpi | 4 dpi | 5 dpi |
| --- | --- | --- | --- |
| CK | 0.00 (0.00) | 0.00 (0.00) | 0.00 (0.00) |
| BZ-FL‑1 | 1.125 (0.40) | 1.625 (0.55) | 2.40 (1.00) |
| BZ-FL‑11 | 1.00 (0.15) | 1.375 (0.40) | 1.75 (0.80) |
| BZ-GF‑1 | 1.05 (0.20) | 1.50 (0.30) | 1.80 (0.70) |
| BZ-GF‑2 | 1.125 (0.20) | 2.20 (0.80) | 3.10 (1.20) |

Note: Data are presented as median (interquartile range, IQR); n=6 per treatment per time point. CK values are all zero; IQR = 0.

Supplementary Table 4. Kruskal‑Wallis test results and significant pairwise comparisons (Dunn’s test with Bonferroni correction) for lesion diameters on *Syzygium samarangen*se at 3, 4, and 5 days post‑inoculation (dpi).

Only comparisons with adjusted *p* < 0.05 are shown. n=6 per treatment per time point.

| Time point | Overall Kruskal‑Wallis | Significant pairwise comparisons (adjusted *p*) |
| --- | --- | --- |
| 3 dpi | χ²(4) = 16.545, *p*=0.002 | CK vs BZ-GF‑1 (0.046); CK vs BZ-FL‑1 (0.010); CK vs BZ-GF‑2 (0.003) |
| 4 dpi | χ²(4) = 22.634, *p*<0.001 | CK vs BZ-FL‑1 (0.013); CK vs BZ-GF‑2 (< 0.001); BZ-FL‑11 vs BZ-GF‑2 (0.046) |
| 5 dpi | χ²(4) = 21.595, *p*<0.001 | CK vs BZ-FL‑1 (0.004); CK vs BZ-GF‑2 (< 0.001) |

Note: Non-significant comparisons (e.g., CK vs FL‑11, CK vs GF‑1 at 4 and 5 dpi, BZ-FL‑1 vs BZ-GF‑2, etc.) are not listed; all had adjusted *p* > 0.05.
